# Assessment of the Therapeutic Potential of Melatonin for the Treatment of Osteoporosis Through a Narrative Review of Its Signaling and Preclinical and Clinical Studies

**DOI:** 10.3389/fphar.2022.866625

**Published:** 2022-05-11

**Authors:** Yongchao Zhao, Guoxi Shao, Xingang Liu, Zhengwei Li

**Affiliations:** Department of Orthopedics, The Second Hospital of Jilin University, Changchun, China

**Keywords:** melatonin, osteoporosis, bone formation, bone resorption, receptor activator of nuclear kappa B ligand

## Abstract

Melatonin is a bioamine produced primarily in the pineal gland, although peripheral sites, including the gut, may also be its minor source. Melatonin regulates various functions, including circadian rhythm, reproduction, temperature regulation, immune system, cardiovascular system, energy metabolism, and bone metabolism. Studies on cultured bone cells, preclinical disease models of bone loss, and clinical trials suggest favorable modulation of bone metabolism by melatonin. This narrative review gives a comprehensive account of the current understanding of melatonin at the cell/molecular to the systems levels. Melatonin predominantly acts through its cognate receptors, of which melatonin receptor 2 (MT2R) is expressed in mesenchymal stem cells (MSCs), osteoblasts (bone-forming), and osteoclasts (bone-resorbing). Melatonin favors the osteoblastic fate of MSCs, stimulates osteoblast survival and differentiation, and inhibits osteoclastogenic differentiation of hematopoietic stem cells. Produced from osteoblastic cells, osteoprotegerin (OPG) and receptor activator of nuclear factor kappa B ligand (RANKL) critically regulate osteoclastogenesis and melatonin by suppressing the osteoclastogenic RANKL, and upregulating the anti-osteoclastogenic OPG exerts a strong anti-resorptive effect. Although the anti-inflammatory role of melatonin favors osteogenic function and antagonizes the osteoclastogenic function with the participation of SIRT signaling, various miRNAs also mediate the effects of the hormone on bone cells. In rodent models of osteoporosis, melatonin has been unequivocally shown to have an anti-osteoporotic effect. Several clinical trials indicate the bone mass conserving effect of melatonin in aging/postmenopausal osteoporosis. This review aims to determine the possibility of melatonin as a novel class of anti-osteoporosis therapy through the critical assessment of the available literature.

## Introduction

Melatonin (Mel) is a bioamine (N-acetyl-5-methoxytryptamine) produced primarily in the pineal gland that critically regulates the sleep–wake cycle. In addition, Mel regulates diverse functions, including seasonal reproduction, immunity, protection of retinal pigment epithelial cells against oxidative damage, and glucose homeostasis (Amaral and Cipolla-Neto, 2018). Mel is also produced in the gut and other peripheral tissues (Jones et al., 2012; Soderquist et al., 2015). It is a highly conserved molecule synthesized in proteobacteria and cyanobacteria. As these bacteria became part of the mitochondria of eukaryotes through endosymbiosis and retained the ability to synthesize melatonin, all organisms (plants and animals) produce this hormone albeit with variations in synthetic pathways (Zhao et al., 2019). Mel is highly soluble in both lipid and water and thus easily diffuses through the cell membrane and the blood–brain barrier (Yu et al., 2016). It has a half-life of about 30 min and is metabolized in the liver and excreted through urine as 6-sulfatoxymelatonin that serves as a surrogate of circulatory Mel levels (Paakkonen et al., 2006; Gooneratne et al., 2012).

Mel signals through G protein-coupled receptors (GPCR), including melatonin receptor 1 (MT1R) and melatonin receptor 2 (MT2R); however, the free radical scavenging effect of Mel is independent of the receptors (Tan et al., 2007; Cecon et al., 2018). GPCRs play a critical role in bone homeostasis. There are 92 GPCRs known to be associated with bone diseases and dysfunction, out of which 36 cause diseases in humans and 72 in animals (Luo et al., 2019). The type 1 receptor for parathyroid hormone (PTH1R), a GPCR upon activation by PTH and PTH-related protein (PTHrP), acts on osteoblasts to stimulate bone formation (Trivedi et al., 2010). On mesenchymal stem cells (MSCs), PTH stimulates the formation of osteoblasts and suppresses adipocyte formation concomitantly (Rickard et al., 2006). Given the bone anabolic effect of PTH1R, the N-terminal fragments of PTH (teriparatide) and PTHrP (abaloparatide) are in clinical use for treating postmenopausal osteoporosis to reduce the risk of spine and hip fractures (Bhattacharyya et al., 2019). Other hormones that signal *via* the GPCRs and regulate bone homeostasis include follicle-stimulating hormone and norepinephrine, and both cause bone loss (Takeda et al., 2002; Sun et al., 2006). In contrast, the thyroid-stimulating hormone stimulates bone mass by suppressing bone remodeling (Abe et al., 2003).

Bone remodeling is the central regulatory event that underlies bone homeostasis in adult mammals and is required to replace old and damaged bone with new bone. The active bone remodeling cycle occurs in all weight-bearing bones, including the lumbar spine, femur, and tibia. This cycle broadly has four stages: 1) activation, when osteoclasts are activated and resorb bone to form a resorption pit; 2) reversal, after the completion of resorption when osteoblast precursors are recruited to the resorption pit; 3) formation, when osteoblasts deposit bone mineral and matrix to fill the pit; and 4) quiescent, when the viable osteoblasts after filling the pit become lining cells and osteocytes to regulate calcium homeostasis and mechanosensing, respectively (Feng and McDonald, 2011). Under physiologic conditions with normal gonadal function, bone remodeling is balanced; in other words, the removal of the old/damaged bone is replaced almost by the same amount of new bone. However, when gonadal function declines, which is defined by the decrease in sex steroid levels, as in cases of menopause and andropause, the bone remodeling cycle displays an imbalance with greater bone resorption than formation, which leads to net bone loss. When net bone loss becomes uninhibited, it gives rise to osteoporosis with the consequent increase in fracture risk (Manolagas et al., 2002).

Available therapies for osteoporosis are classified under remodeling suppressors (anti-resorptive), remodeling enhancers (bone anabolic), and mixed (both anti-resorptive and bone anabolic) (Langdahl, 2021). Bisphosphonates, selective estrogen receptor modulators, and neutralizing antibody against RANKL are anti-resorptive, of which bisphosphonates are the first-line therapy of osteoporosis (Langdahl, 2020). Teriparatide and abaloparatide are bone anabolic drugs prescribed to patients with a high risk of osteoporotic fracture (Trivedi et al., 2010; Bhattacharyya et al., 2019). A neutralizing antibody against sclerostin (romosozumab) is claimed to be both anti-resorptive and bone anabolic. However, this has not been attested in clinical trials. Romosozumab is considered a bone anabolic like PTH1R targeted drugs. Although bone anabolic therapy is most desirable to restore the lost bone, given its mode of action on the remodeling cycle, which is to stimulate both formation and resorption, the anabolic effect is lost after an initial phase of increase (Bhattacharyya et al., 2018). Therefore, a therapy that stimulates bone formation without increasing resorption or suppressing it would represent a major advancement over the existing anti-osteoporosis therapy.

Mel may possess both bone-forming and anti-resorptive effects, as shown by *in vitro* and preclinical animal studies. Herein, we discuss the pharmacology of Mel receptors in general and the effect of Mel on bone cells, its downstream signaling, skeletal effects in preclinical models of bone loss, and clinical studies assessing its skeletal impact in aging and postmenopausal subjects.

## Pharmacology of Melatonin Receptors

MT1R and MT2R have considerable sequence homology in the transmembrane region (Reppert et al., 1994). Both receptors are mainly coupled to Gα_i/o_ proteins, and, consequently, decrease intracellular levels of the second messenger cAMP, which is the most commonly observed signaling pathway activated by Mel (Dubocovich et al., 2010). Additional intracellular cascades activated by Mel include MEK/ERK kinases and the recruitment of β-arrestins (Chen M. et al., 2020). GPR50 is an orphan receptor with 50% sequence homology with MT1R and MT2R but does not bind with Mel or any other known ligands (Reppert et al., 1996).

**FIGURE 1:**
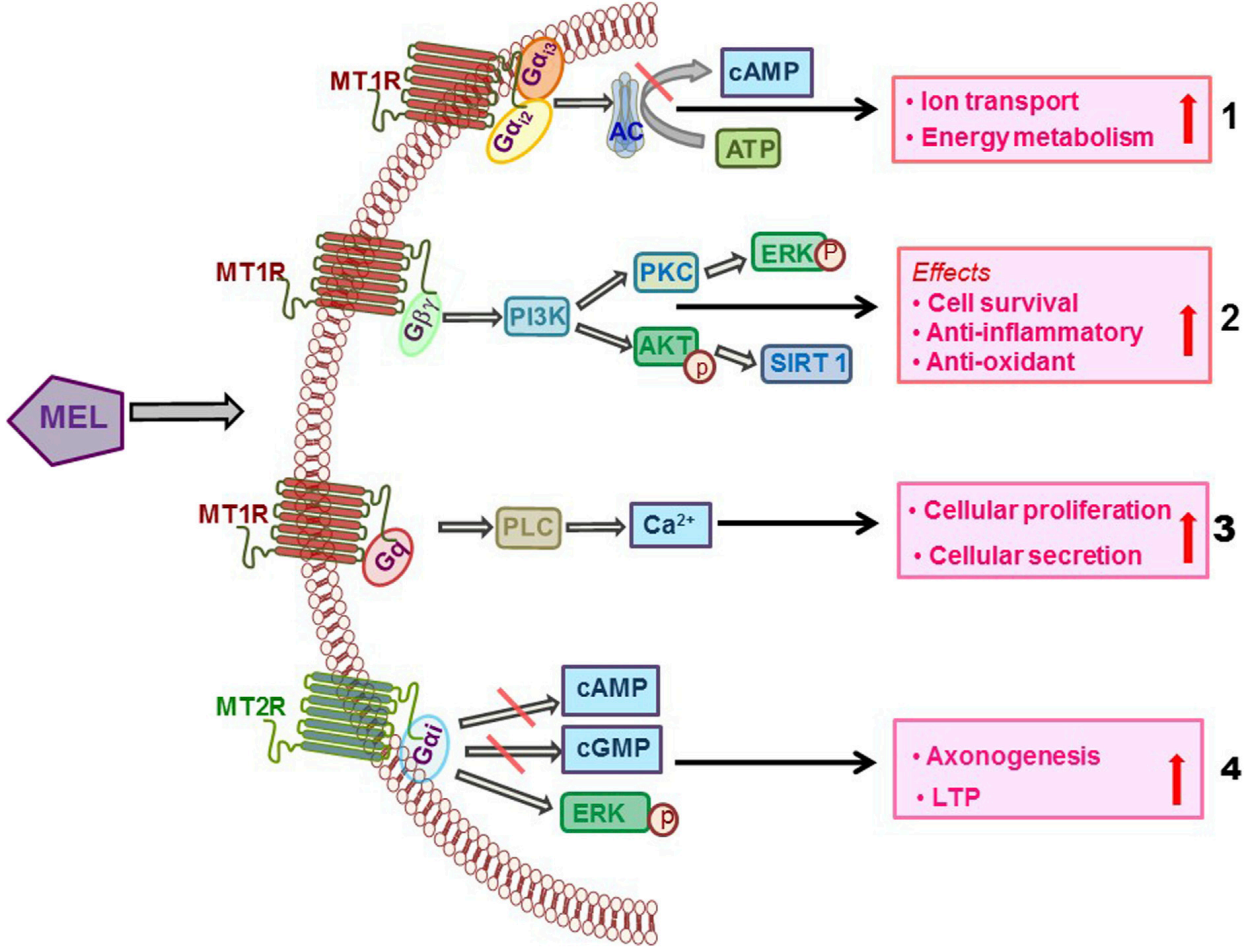
Diverse coupling of Mel receptors with G proteins and consequent activation of the downstream cellular signaling that mediates the effects of Mel.

In HEK-293 cells, MT1R co-immunoprecipitated preferentially with Gα_i2_ and Gα_i3_ proteins and to a lesser extent with G_q/11_, but not with Gαi_1_, Gα_z_, Gα_o_, Gα_12_, or Gα_s_, which suggested that MT1R is coupled with Gα_i2_ and Gα_i3_ proteins (Brydon et al., 1999). Mel activated the JNK pathway when MT1R or MT2R was co-transfected with Gα_16_ in COS-7 cells, indicating coupling of both melatonin receptors to Gα_16_ (Chan et al., 2002). Coupling of MTRs with Gq/11 has been reported in the myometrium (Sharkey et al., 2010), MSCs (Lee et al., 2014), prostate epithelial cells (Shiu et al., 2010), and pancreatic cells (Bahr et al., 2012), and the consequent downstream events include production of diacylglycerol, inositol trisphosphate, and intracellular rise in Ca^2+^ levels. The heterogeneity of coupling of Mel receptors with G proteins and consequent modulation of cellular events is shown in Figure 1.

**FIGURE 2:**
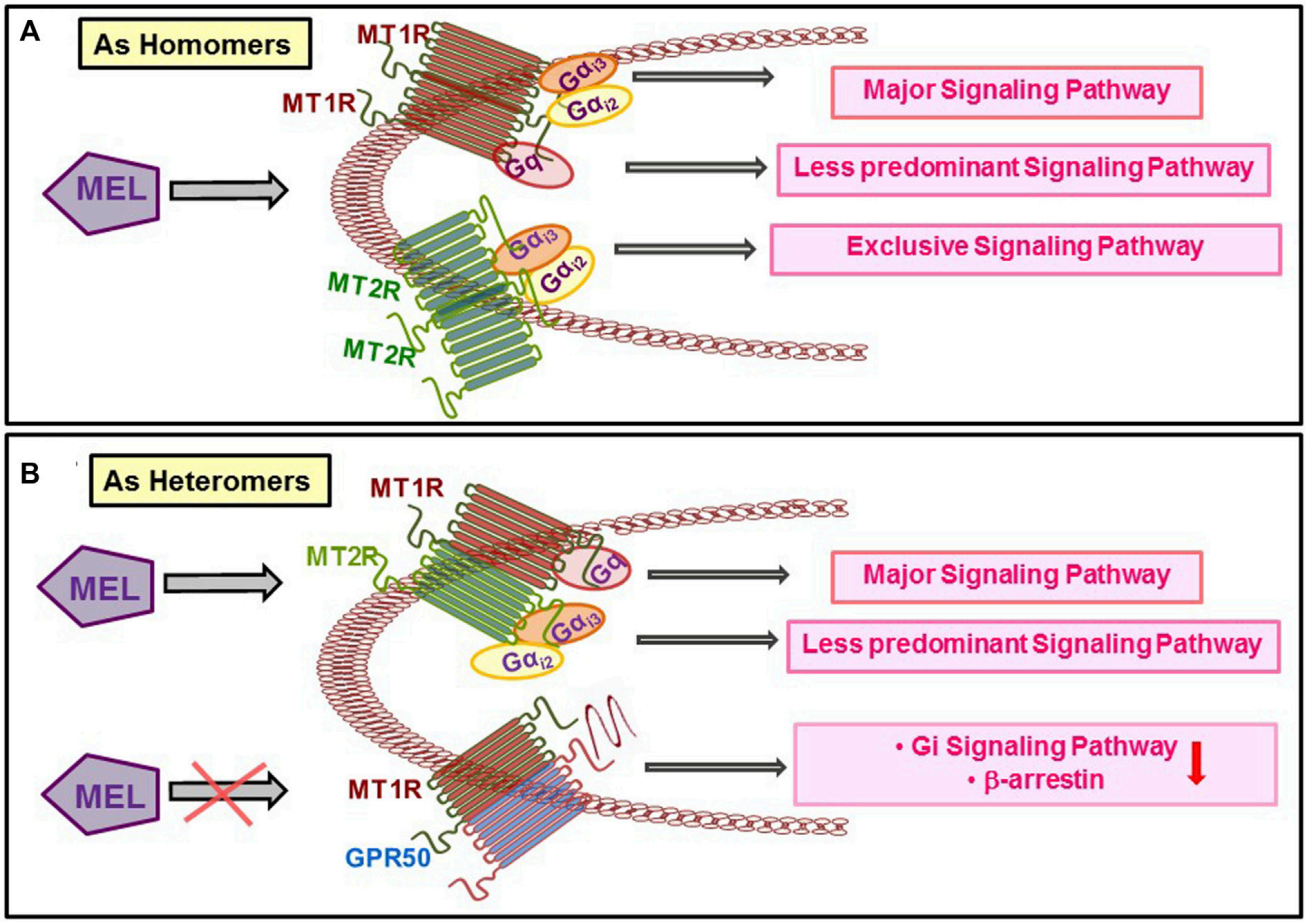
Dimerization of the different types of Mel receptors regulates the preference for G protein binding and signalling.

At their physiological expression levels, human MT1R and MT2R have a high potential to homo- and heteromerize in a constitutive fashion (Ayoub et al., 2002). The possibility for homo- and heteromer formation is variable as MT1R homomer, and MT1R–MT2R heteromer formations are much greater than MT2R heteromer (Ayoub et al., 2004). The functional outcome of MT1R and MT2R heteromer is mostly unknown except that such event occurs in retinal rod cells and activates PLC/PKC pathway (Baba et al., 2013). Current evidence strongly suggests that the signaling by the Mel receptors is highly cell- and tissue-dependent, supporting the existence of system bias regulating the functional outcome that is further dependent on the differences in the expression of receptor-associated proteins, including the formation of homo- and heteromeric receptors. G proteins coupling selectivity of Mel receptors *vis-à-vis* dimerization is shown in Figure 2.

There are several small molecule antagonists and agonists used to understand the pharmacologic actions of Mel in finer detail. Luzindole (N-acetyl-2-benzyltryptamine) is a competitive non-selective receptor antagonist widely used to examine the membrane effect of Mel. 4-Phenyl-2-propionamidotetralin (4P-PDOT) is an MT2-selective antagonist used to discriminate the effects between MT1R and MT2R. A specific MT1R antagonist is not yet available. The agonist of Mel receptors includes ramelteon ((S)-N-[2-(1,6,7,8-tetrahydro-2H-indeno-(5,4-b)furan-8-yl)ethyl]propionamide), agomelatine (N-[2-(7-methoxynaphthalen-1-yl)ethyl]acetamide), and tasimelteon (*N*-[[(1*R*,2*R*)-2-(2,3-dihydro-1-benzofuran-4-yl)cyclopropyl]methyl]propanamide). All agonists are non-selective and have similar affinities for human MT1R and MT2R (Millan et al., 2003; Kato et al., 2005). Despite being non-selective, ramelteon has a higher affinity for MT1R over MT2R (Kato et al., 2005), and agomelatine and tasimelteon show a higher affinity to MT2R over MT1R (Lavedan et al., 2015). Of these agonists, ramelteon is used for the treatment of insomnia and the other two are used for the treatment of sleep and circadian disturbances.

## Effect of Melatonin on Bone Cells and Associated Signaling

In human adult MSCs, functional melatonin receptor was demonstrated by radioreceptor assay, and upon the induction of osteogenic differentiation, the binding of melatonin and its differentiation promoting function were increased. In these cells, Mel stimulated osteogenic differentiation by transactivation of epidermal growth factor receptor (EGFR) through the activation of metalloproteinase, which resulted in the downstream activation of MEK1-Erk1/2, leading to osteogenic differentiation (Radio et al., 2006). ROS accumulation results in oxidative damage to mitochondrial function and contributes to the etiology of osteoporosis. In human MSCs, Mel restored H_2_O_2_-mediated oxidative stress-inhibited osteoblast differentiation by activating AMPK signaling, which then activated FOXO3a and Runx2, the master osteogenic transcription factor (Lee et al., 2018). When human peripheral blood mononuclear cells (PBMCs, precursor of osteoclasts) were exposed to Mel, oxidative stress was significantly mitigated with the attendant restoration of manganese superoxide dismutase (MnSOD) activity (Emamgholipour et al., 2016). Similar to the findings in MSCs, Mel ameliorated H_2_O_2_-induced oxidative stress in MG-63, human osteosarcoma cells, and maintained mitochondrial ATP production and mitochondrial function (She et al., 2014). Moreover, Mel also inhibits PPARγ expression in MSCs, thereby suggesting that it favors osteogenic over adipogenic differentiation in bone marrow (Maria et al., 2018) and confirming the findings of Zhang et al. (2010). Accumulation of fatty acids, particularly triglycerides (TG) in osteoblast precursors, inhibits osteogenic differentiation and switches these cells to adipocytes (Diascro et al., 1998). Mel inhibits TG accumulation in osteoblasts (ROS17/2.8 cells) with or without oleic acid (Sanchez-Hidalgo et al., 2007).

Inflammation is known to inhibit osteoblast functions. Inflammation diseases, including IBD, RA, and COPD, have been associated with osteoporosis (Hardy and Cooper, 2009). One of the major inflammatory cytokines that become abundant in the blood is tumor necrosis factor alpha (TNFα). Mel protected BMSCs from TNFα-induced ROS generation, reductions in osteogenic differentiation by upregulating antioxidases (SOD, catalase, and glutathione) and downregulation of oxidases (NADPH oxidases 1 and 2). Furthermore, Mel phosphorylates p65 protein and blocks the degradation of inhibitor of κΒα (IκΒα), resulting in the reduced activity of the nuclear factor kappa B (NF-κB) pathway (Qiu et al., 2019), which favors osteogenic differentiation. Mel also suppresses the function of NF-kB action by upregulating Wnt4 by the ERK1/2-Pax2-Egr1 pathway. Increased production of Wnt4 has an osteogenic effect through the canonical Wnt-β-catenin and non-canonical Wnt-p38-JNK pathways. The canonical pathway activation by Mel was mediated by Wnt4-Fzd1-LRP5 and Wnt4-Fzd6-LRP6, whereas the p38-JNK pathway was mediated by Wnt4-Fzd2 interaction (Li X. et al., 2019). In addition to mitigating the activity of the NF-κB pathway (Qiu et al., 2019), Mel also rescued the attenuation of TNFα-induced SMAD-specific E3 ubiquitin protein ligase 1 (SMURF1) expression that then protected SMAD1 protein from being degraded by the SMURF1-mediated ubiquitination, resulting in the maintenance of bone morphogenetic protein (BMP) SMAD1-mediated osteogenic signaling in MSCs (Lian et al., 2016).

Mel potentiated the function of osteogenic growth factor, that is, BMP-4. In the C2C12 pre-myoblast cell line, Mel alone did not stimulate osteoblast differentiation. However, in the presence of BMP-4, Mel stimulated osteogenic differentiation by increasing osterix, a zinc finger containing transcription factor that promotes osteoblast differentiation in a Smad-dependent mechanism. One of the mechanisms of Mel-mediated increase in osterix expression involved stabilization of osterix protein by the inhibition of the ubiquitin–proteasome pathway. Moreover, in C2C12 cells, both PKA and PKC pathways are involved in the transactivation of osterix by Mel (Han et al., 2017).

Mel has been reported to regulate osteogenic differentiation by a transcriptional mechanism involving miRNA and circular RNA. In mouse bone marrow stromal cells, Mel stimulates osteogenic differentiation by increasing the expression of miR-92b-5p that directly targets intracellular adhesion molecule-1 (ICAM-1). Because ICAM-1 inhibits osteogenesis of BMSCs, Mel-induced upregulation of miR-92b-5p serves to augment osteogenesis. Moreover, miR-92b-5p is downregulated in the BMSCs of osteopenic mice, and the resultant impairment of osteoblast differentiation of these cells in response to Mel could be rescued by overexpressing miR-92b-5p (Li Y. et al., 2019). Human BMSCs, upon treatment with Mel followed by deep RNA sequencing, identified a circular RNA, circ_0003865, that was repressed by Mel. circ_0003865 sponges miR-3653-3p, which suppresses growth arrest specific-1 (GAS1) protein to enhance osteogenic differentiation of BMSCs. Hence, by repressing circ_0003865, Mel favors osteogenic differentiation. These *in vitro* observations were confirmed in OVX mice, where sh_circ_0003865 delivery by AAVs protected against the development of osteopenia by upregulating miR-3653-3p (Wang et al., 2021).

In addition to affording protection against inflammation and ROS-induced suppression of osteoblastic cells, Mel protects osteoblasts against glucocorticoid (GC)-induced and glucose-induced suppression of differentiation. In MC3T3-E1 murine osteoblasts, Mel prevented GC (dexamethasone)-mediated inhibition of differentiation *via* the proximal phosphoinositide-3-kinase (PI3K)/Akt (protein kinase B) and downstream BMP/SMAD pathway (Zhao et al., 2020). Moreover, Mel prevented glucotoxicity-induced osteoblast apoptosis by attenuating endoplasmic reticulum (ER) stress by regulating PERK–eIF2α–ATF4-CHOP signaling. Mel action was mediated by both MT1R and MT2R (Zhou R. et al., 2020). Elucidation of this mechanism is important as oxidative stress resulting in the generation of free radicals has been linked to ER stress in diabetic patients, ultimately leading to loss of osteoblast population due to apoptosis (Burgos-Moron et al., 2019). Ferroptosis is a novel type of programmed cell death that impairs glucose-stimulated insulin secretion by damaging pancreatic β-cells as these cells are vulnerable to oxidative damage due to the lack of a strong antioxidant defense mechanism (Sha et al., 2021). Table 1 gives the summary of various events in osteoblasts that Mel regulates.

**TABLE 1:** Osteogenic pathways and molecular mediators.

Cellular event	Signaling mechanisms	References
Stimulation of differentiation	EGFR transactivation and Mek-Erk1 activation; activation of AMPK signaling followed by the upregulation of FOXO3a and Runx2; increase in miR-92b-5p and inhibition of ICAM-1; downregulation of circ_0003865; suppression of GAS1	Radio et al. (2006), Lee et al. (2018), Li et al. (2019b), Wang et al. (2021)
Inhibition of bone marrow adipogenesis	Downregulation of PPARγ; upregulation of lnc RNA H19 to spongemiR-541-3p	Maria et al. (2018), Han et al. (2021)
Protection against ROS and inflammation	Upregulation of antioxidases; activation of canonical and non-canonical wnt pathway; inhibition of NF-κB pathway; attenuation of SMURF1 and maintenance of BMP-Smad1 signaling; suppression of Erk activation	Emamgholipour et al. (2016), Qiu et al. (2019), Li et al. (2019a), Lian et al. (2016), Zhang et al. (2016)
Protection against glucotoxicity	Attenuation of ER stress through PERK–eIF2α-ATF4-CHOP signaling; attenuates senescence by downregulating p16, p21, p53, and γH2AX; upregulation of Sirt-1; activation of Nrf2-HO-1 pathway	Zhou et al. (2020a), Gong et al. (2021), Ma et al. (2020a)
Protection against glucocorticoid-induced osteoblast differentiation	Activation of PI3K/Akt and BMP-Smad signaling	Zhao et al. (2020)

In addition to suppressing osteoblast viability and differentiation, oxidative stress favors osteoclastogenesis. Given that Mel protects osteoblasts and osteoblast precursor cells against apoptosis and inhibition of differentiation, it is surmised that Mel could inhibit osteoclastic differentiation of the precursor cells. Mel inhibits osteoclastogenesis of bone marrow macrophages at the pharmacological concentrations (1–100 μM) but not at the physiological concentration (0.1–10 nM). The mechanism appears to be the suppression of receptor activator of NF-κB ligand (RANKL)-induced ROS production by bone marrow macrophage (BMM) through the inhibition of NF-κB activation. Unlike the involvement of silent information regulator-1 (SIRT-1) in osteogenic differentiation of MSCs, the inhibition of osteoclastogenesis by Mel was SIRT-1 independent (Zhou et al., 2017). In a murine monocyte/macrophage cell line, RAW264.7 Mel first decreases miR-882 expression that results in the increase of the expression of transcription regulator of the circadian clock Rev-erbα and decreases cathepsin K expression, ultimately inhibiting osteoclast formation and function (Tian et al., 2021). In a co-culture system using human MSCs and PBMCs, Mel *via* MT2R increased osteoblastogenesis and decreased osteoclastogenesis by increasing osteoprotegerin (OPG) and decreasing the OPG: RANKL ratio. The underlying mechanism involved the modulation of pERK1/2 pERK5, integrin-β1, NFκB, and GLUT4. In addition to upregulating the anti-osteoclastogenic cytokine, OPG production from osteoblasts, Mel directly inhibits osteoclastogenesis (Maria et al., 2018). These reports suggest that Mel suppresses osteoclastogenesis by both direct and indirect mechanisms.

Studies on the effects of Mel on bone cells suggest that the hormone has multiple salutary effects, including stimulation of osteogenic differentiation, an increase in osteoblast survival, and inhibition of adipocyte and osteoclast formation and is likely to culminate in protecting bones against the development of osteoporosis.

## Skeletal Effects of Melatonin in Preclinical Disease Models

### Pinealectomy and the Role of Melatonin

Scoliosis is a condition characterized by deformity in the lumbar and thoracic spine and causes osteopenia or osteoporosis in young individuals (Sadat-Ali et al., 2008). Experimental scoliosis can be modeled in chickens by pinealectomy (PNX). Newly hatched chicks given Mel for 8 weeks show an increase in bone accrual and better microarchitecture in the spine. PNX of newly hatched chicken and maintained for 8 weeks resulted in vertebral (scoliotic) deformity, decreased length and weight of the vertebral bodies of the spinal column, and reduced spinal BMD compared with non-PNX chicks (Turgut et al., 2005). Similar to that in the ovariectomized (OVX) condition, PNX results in high turnover bone loss characterized by increased osteoblast and osteoclast surface and number that accompanied decreased trabecular bone volume and poor microarchitecture when mid-portion of vertebrae was studied (Aota et al., 2013). Mel treatment to PNX chicks maintained bone volume, trabecular microarchitecture, and osteoblast number to the sham level. However, the osteoclast number in the bones was not altered by PNX (Kono et al., 2011). From these data, Mel derived from the pineal gland contributes to skeletal homeostasis in birds.

Deleting the pineal gland-specific gene, tryptophan hydroxylase (Tph1), the enzyme in the synthetic pathway of Mel results in mice deficient in this hormone. These mice displayed a low bone mass phenotype due to a defect in bone formation. Furthermore, mice lacking MT2R and not MT1R displayed a low bone mass phenotype caused by reduced osteoblast proliferation and differentiation (Sharan et al., 2017). The effect of PNX in a large animal was studied in sheep. Static histomorphometry measurements at the iliac crest biopsy showed equivalent loss of trabecular bones in PNX and OVX animals. Bone resorption markers measured by collagen degradation products, including serum pyridinoline and urinary deoxypyridinoline, increased transiently at 3 and 6 months after PNX (Egermann et al., 2011).

### Ovariectomy and the Effect of Melatonin

Bilateral ovariectomy removes the source of estrogen and is a widely used model for postmenopausal osteoporosis. When ovariectomy is performed in laboratory rodents, similar to postmenopausal women, it induces a rapid trabecular bone loss followed by cortical bone loss, ultimately resulting in loss of bone mechanical strength (Kushwaha et al., 2014). Furthermore, bone turnover markers, including the bone resorption and formation markers in OVX rodents a similar trend as in postmenopausal women with a rise in the resorption markers. However, bone formation markers were initially elevated but decline with time (Szulc et al., 2017; Pal et al., 2020).

In OVX mice, Mel had a dual favorable action of inhibiting bone resorption and stimulating bone formation, resulting in complete restoration of trabecular bone mass at a 100 μM dose. This effect was achieved by increasing hepatocyte growth factor (HGF) production from BMSCs, which then activated the osteogenic Wnt-β-catenin pathway by downregulating phosphatase and tensin homolog (PTEN). The mediatory role of HGF has been elegantly shown by infusing si-HGF to OVX mice treated with Mel, which showed abolition of the bone-promoting effect of the hormone. Mel also upregulated various osteogenic molecules, including BMP-2, BMP-4, osteocalcin, Runx2, and sp2 (Zhang et al., 2021). In OVX mice, Zhou Y. et al. (2020) reported the anti-osteoporotic effect of Mel at a 10 mg/kg dose but not at 100 mg/kg, similar to the case with the osteogenic serum marker, procollagen type 1 N-terminal propeptide (P1NP); diminished OVX level compared with sham was increased by only the lower dose of Mel. However, at the higher dose of Mel, the expression levels of osteogenic genes in bones, including Runx2, osterix, type I collagen, osteocalcin, and alkaline phosphatase (ALP), were significantly increased over the corresponding OVX levels. Osteoclastogenic markers in the blood, including type I collagen cross-linked C-telopeptide (CTX-1) and tartrate-resistant acid phosphatase (TRAP), were completely decreased by the higher Mel dose similar to that of the lower dose. The underlying mechanism appears to be the downregulation of inflammatory response in bone marrow MSCs resulting in higher OPG-to-RANKL production that in turn leads to a decrease in osteoclastogenesis. Given the equivalent decrease in osteoclastogenic response and increase in the osteoblastogenic response by lower and higher doses of Mel in OVX mice, a lack of improvement in bone mass and volume in the higher dose of Mel group is incomprehensible (Zhou Y. et al., 2020). The anti-resorptive effect of Mel is observed in estradiol (E2) deficient but not in the E2 replete condition. Accordingly, in ovary intact rats, Mel did not inhibit the serum CTX-1, but it did in OVX rats. Although this effect was lesser than E2, combining Mel with E2 had an additive effect as CTX-1 was suppressed more than E2 or Mel-lone treatment given to OVX rats. This additive effect translated into the skeletal response as the spine and tibial bone area and whole-body bone mass were significantly higher than either hormone (Ladizesky et al., 2003).

E2 deficiency, such as that after menopause, results in the increased formation of adipocytes from the bone marrow MSCs at the expense of osteoblasts. As a result, bone formation is inhibited while osteoclast-stimulating cytokines from adipocytes are increased, and together these two events contribute to bone loss. Canonical Wnt pathway through β-catenin has reciprocal effects, favoring osteogenesis and inhibiting adipogenesis. Accordingly, in OVX rats, bone marrow adipogenesis is increased with a concomitant decrease in osteogenesis, and Mel activated the canonical Wnt pathway to reverse these events and restore bone mass that involves complex participation of lnc RNA, mi-RNA, and Wnt pathways. In this regard, Han et al. (2021) showed that Mel upregulated lnc RNA H19 (having an osteogenic effect) that sponged miR-541-3p (having an adipogenic effect), caused a decrease in the adipogenic differentiation, and enhanced the osteogenic differentiation of bone marrow MSCs. Since miR-541-3p targets adiponectin, its downregulation upregulates adiponectin levels in BMSCs. Given the osteogenic effect of adiponectin (China et al., 2017; Pal China et al., 2018), it is surmised that, by favorably regulating the H19-miR-541-3p-adiponectin axis, Mel promoted bone formation in osteopenic rats.

The levels of inflammatory cytokines are increased after menopause. One of the major mediators of inflammatory cytokines in bone is the nucleotide-binding domain and the leucine-rich repeat pyrin 3 domain (NLRP3) inflammasome, which, upon activation, inhibits osteogenic differentiation and favors adipogenic differentiation of MSCs (Wang et al., 2017). In OVX femoral bones, the levels of NLRP3 components, including NLRP3, apoptosis-associated speck-like protein containing CARD (ASC), pro-caspase-1, caspase-1 (p10), pro-IL-1β, and active IL-1β, were increased over the sham, and Mel treatment significantly inhibited their levels in the OVX bones. NLRP3 inflammasome signaling inhibits osteogenic differentiation, and Mel stimulated the event by suppressing the activation of the inflammasome by activating the Wnt/b-catenin pathway (Xu et al., 2018).

### Aging-Induced Bone Loss and the Effect of Melatonin

Aging displays loss of bone mass, deterioration of bone microarchitecture, and reductions in biomechanical strength, thereby leading to increased fracture risk (Demontiero et al., 2012). In 20-month aged male rats, Mel (50 mg/kg i.p.) treatment for 12 weeks increased bone mass, improved trabecular microarchitecture, decreased urinary loss of calcium and phosphate, increased the serum osteogenic markers (bone-specific ALP and osteocalcin), increased bone formation rate, increased the osteogenic differentiation, and reduced adipogenic differentiation of bone marrow stromal cells. These data suggested that Mel acts as a bone anabolic hormone in aging-induced bone loss (Chu et al., 2017). In adult mice (4 months), a long duration Mel treatment (up to 20 months) *via* the oral route increased the plasma Mel levels and preserved bone mass and bone strength of the femur to the levels of the adult mice (Igarashi-Migitaka et al., 2020). In aged rats (22 months, equivalent to 60 years of human age), 10-week treatment of Mel protected against age-related loss of bone mass and strength by stimulating osteoblast markers in bone (Tresguerres et al., 2014). These reports suggest that Mel supplementation could inhibit natural aging-induced bone loss.

### Diabetes-Induced Bone Loss and the Effect of Melatonin

Diabetic osteoporosis is a common type of metabolic disease in which bone quality is impaired due to senescence caused by high glucose (Farr et al., 2016; Eckhardt et al., 2020). Mel alleviates osteoblast senescence induced by high glucose (HG) and protects against diabetes-induced bone loss. In murine osteoblastic cell line, MC3T3-E1, HG caused concentration-dependent loss of proliferation, and it was rescued by Mel by reversing the HG-induced increase in cells in the G1 phase and decreased population in the S phase. Mel downregulated the senescence proteins, including p16, p21, p53, and γH2AX, caused by HG by upregulating Sirt-1. Furthermore, *in vivo*, in male mice with type 1 diabetes (induced by streptozotocin), the aforementioned senescence proteins were increased, and Mel treatment mitigated their levels with a concomitant increase in bone mass and improvement of microarchitecture. Sirt-1 was also increased in the bone of diabetic rats treated with Mel, thus suggesting that this NAD+-dependent histone deacetylase has a crucial role in imparting anti-senescence and anti-DNA damage effects in osteoblasts and serves as an important mechanism to protect against bone loss (Gong et al., 2021). HG is also known to cause oxidative stress, which stimulates autophagy in several cell types by activating the Erk pathway (Hung et al., 2016; Mi et al., 2016; Yeh et al., 2016). HG-induced oxidative stress is particularly relevant in diabetes conditions. In a type 2 diabetes rat model induced by a low dose of streptozotocin (30 mg/kg instead of 60 mg/kg), Mel treatment for 12 weeks protected diabetic rats from developing osteopenia with attendant stimulation of osteoblast function by mitigating HG-induced ROS production, autophagy induction, and suppression of Erk activation (Zhang et al., 2016).

HG also induces ferroptosis in osteoblasts assessed by morphological hallmarks such as reduced mitochondrial volume, disappearance of cristea, and downregulation of GPx and cystine glutamate antiporter (SLC7A11). In addition, HG caused lipid peroxidation and reduced osteoblast apoptosis. Mel prevented these events culminated in the mitigation of HG-mediated decrease in osteoblast differentiation. Furthermore, Nrf2-HO-1, the endogenous antioxidant pathway, was suppressed by HG, and Mel reversed this effect. In an insulin-resistant rat model (induced by feeding high fat and high sugar) that represents T2DM, Mel mitigated the development of osteopenia by increasing Nrf-2 (Ma H. et al., 2020).

Bone marrow MSCs (BMMSCs) from osteopenic animals display poor antioxidant defense repertoire, including reduced SOD1, SOD2, GPX1, and SIRT-1, with an attendant impairment in osteoblast differentiation compared with animals with normal bone mass. Mel treatment to osteopenic rats not only preserved the antioxidant machinery in BMMSCs downstream of SIRT-1 but also maintained the bone mass and architecture (Chen W. et al., 2020). Through SIRT3/SOD2 signaling, Mel has been shown to ameliorate mitochondrial oxidative stress to increase osteogenesis and improve bone mass around prostheses, thereby implying that Mel could be useful in total joint arthroplasty for increasing the lifespan and stability of the prostheses (Zhou et al., 2019). Rapamycin, an immunosuppressive drug that induces autophagy by inhibiting mTOR, is a suppressor of autophagy. Rapamycin has been shown to increase bone mass in the senile male osteoporosis model by activating osteocyte autophagy (Luo et al., 2016). In senile female rats, a combination of rapamycin and Mel afforded greater protection of bone mass and strength by favorable modulation of the OPG-to-RANKL ratio through regulation of osteoblast function (Tao et al., 2020).

Emerging data suggest that citrate is an integral component of apatite nanocrystal and accounts for 5.5% (wt%) of the organic matter of the bone. Citrate is a major provider of carboxylate for calcium bonding in the bone and critically contributes to bone strength and resistance to fracture (Costello et al., 2012). In a randomized, double-blind placebo-control trial on healthy elderly persons, supplementation of potassium citrate for 2 years significantly increased BMD and improved microarchitecture over the placebo group (Jehle et al., 2013). OVX rat bones have significantly reduced citrate content than the sham (ovary intact) control, suggesting that citrate is directly related to bone loss. Mel treatment to OVX rats increased the bone citrate content back to the level of the sham. In cultured osteoblasts, Mel stimulated mineralized matrix formation by upregulating Zn^2+^-transporter-1 (ZIP-1), as silencing ZIP-1 abrogated the mineralizing action of Mel. Moreover, ZIP-1 was downregulated in OVX bones, and Mel treatment completely restored it to the sham level (Da et al., 2020).

Gelatine methacryloyl-dopamine (GelMA-DOPA) has been widely used in bone tissue engineering due to its efficient adhesive capability on wet surfaces and biocompatibility (Schuurman et al., 2013; Ma D. et al., 2020; Keri et al., 2020). The success of implanted biomaterials to remain adherent is diminished when the viability of osteoblasts is reduced due to oxidative stress response at the fracture site, mostly due to vasculature. To address the issue, Mel was combined with GelMA-DOPA to fabricate a composite implantation material to stimulate osseointegration in the osteoporotic conditions through the sustained release of Mel. In OVX rats, this composite implant material decreased osteoblast apoptosis caused by oxidative stress and improved bone formation around the prosthesis by signaling through Sirt3/SOD pathway, thus indicating a potential use of Mel in biomaterial implants for accelerated healing of fractures and remaining secure at the fracture site (Xiao et al., 2020).

Table 2 summarizes the skeletal effects of Mel in various preclinical disease models.

**TABLE 2:** Skeletal effects of Mel in preclinical models of bone loss.

Disease model	Effects	References
PNX	a) In newborn chicks, it caused vertebral (scoliotic) deformity and reduced spinal BMD	Turgut et al. (2005), Aota et al. (2013), Egermann et al. (2011)
	b) In young chickens, it caused high turnover bone loss and loss of trabecular microarchitecture in the vertebra	
	c) In adult sheep, it caused trabecular bone loss at the iliac crest equivalent to OVX animals	
Tph1 deletion in the pineal gland	Low bone mass phenotype and exogenous Mel restored bone mass	Sharan et al. (2017)
Mel receptor deletion	MT2R but not MT1R deletion has osteopenic phenotype	Sharan et al. (2017)
OVX	a) In mice, OVX caused bone loss at both tissue and serum marker levels and Mel reversed both	Zhang et al. (2021), Zhou et al. (2020b), Xu et al. (2018)
	b) In rats, OVX caused osteopenia and Mel maintained bone mass	Han et al. (2021), Da et al. (2020)
Prosthesis model developed in OVX rats	Mel in a composite hydrogel system was applied at the distal femur around titanium implant for the sustained release of the hormone, resulting in the increased osteogenesis around prosthesis	Xiao et al. (2020)
Aging	a) In 20-month-old rats, Mel treatment for 12 weeks increased bone mass and bone formation markers	Chu et al. (2017)
	b) In 22-month-old rats, Mel treatment for 10 weeks preserved bone mass and strength	Tresguerres et al. (2014)
	c) Long duration Mel (starting at 4 months until 20 months) maintained bone mass equivalent to adult animals	Igarashi-Migitaka et al. (2020)
Streptozotocin-induced diabetes	Mel protected diabetes-induced bone loss	Gong et al. (2021), Zhang et al. (2016)

## Clinical Assessment of Melatonin in Diseases of Bone Loss

Diurnal changes are known to affect bone metabolism as studies suggest a negative correlation of BMD (Kawabata, 1990), increased association of fracture (Feskanich et al., 2009), and increased bone turnover markers (Vasikaran et al., 2011) with circadian disruption by long-term night shift work. Although none of the studies report Mel levels, an *a priori* decrease in Mel levels contributing to impaired bone response in the night-shift workers can be considered the cause. These human studies were sufficiently compelling to investigate the effect of Mel in diseases of bone loss.

In postmenopausal osteopenic women (*n* = 11), Mel (5 mg) in combination with 450 mg strontium (citrate), 2000 IU vitamin D3, and 60 μg vitamin K2 (MSDK) favorably modulated bone remodeling and increased BMD at the lumbar spine and femur neck compared to the placebo group. Women treated with MSDK also showed a decrease in serum CTX-1 and an increase in serum P1NP. Although women in the MSDK arm had higher urinary melatonin sulfate than the placebo group, whether the positive skeletal effect of MSDK was solely due to Mel cannot be ascertained (Maria et al., 2017). A highly enriched population of BMMSCs (CD34−/CD31−) from young and old women was obtained, and a transcriptome profile was carried out. Mel pathway was altered in BMMSCs of older women along with mTOR, gap junction, calcium, and NFAT signaling pathways, which indicates an important function of Mel in age-associated loss of osteoblast population in the bone marrow, which is an important cause of osteoporosis (Roforth et al., 2015).

In CKD patients undergoing hemodialysis (HD), Mel was reduced in those with osteoporosis than in those who are not. In addition to reduced Mel, HD patients with osteoporosis display higher serum inflammatory cytokines, including TNFα, IL-1, and IL-6, and oxidative stress markers, including higher advanced oxidation protein products and malondialdehyde produced by the peroxidation of unsaturated fatty acids in the cell membrane due to superoxide anion in the body. This study concludes that inflammatory cytokines and oxidative stress markers are negatively correlated, and Mel is positively correlated with BMD (Ren et al., 2018).

In a pilot randomized study, perimenopausal women (*n* = 13) given nightly Mel (3 mg p.o.) for 6 months had no significant effect on BMD and bone turnover markers (NTX and osteocalcin) while improved menstrual cycling (mean cycles and duration of menses) (Kotlarczyk et al., 2012). In a double-blind RCT, Mel (1 and 3 mg p.o.) dose-dependently increased femur neck areal BMD (assessed by DXA) after 1-year treatment. In addition, trabecular thickness at the tibia measured by high-resolution peripheral quantitative computed tomography (HRpQCT) was increased in the (combined) Mel group compared with placebo. Simulated failure load assessed by finite element analysis of HRpQCT images showed no effect of Mel treatment on tibia and radius. None of the BTMs, including osteocalcin, P1NP, CTX-1, and BSAP, was different between the Mel and the placebo groups. There was a significant decrease in urinary calcium in the Mel group compared with the placebo, which could be explained by the increased osteogenic effect of Mel, resulting in increased mineralization (Amstrup et al., 2015).

Girls with anorexia nervosa (AN) have significantly higher nocturnal Mel accompanied by a significantly decreased OPG/RANKL ratio compared with healthy age- and sex-matched control suggesting increased resorption and bone turnover rate to be the possible outcomes of AN. Surprisingly, the bone turnover markers, including CTX-1 and osteocalcin, were both suppressed in AN compared with control. Osteocalcin and CTX-1 levels were, respectively, >300% and 13% reduced in AN, reflecting a disproportionate suppression of osteoblast function in AN. Because mechanical loading positively contributes to osteoblast differentiation, a ∼30% decrease in the body weight in girls with AN could cause reduced osteocalcin levels (Ostrowska et al., 2010).

## Summary and Future Perspectives

**FIGURE 3:**
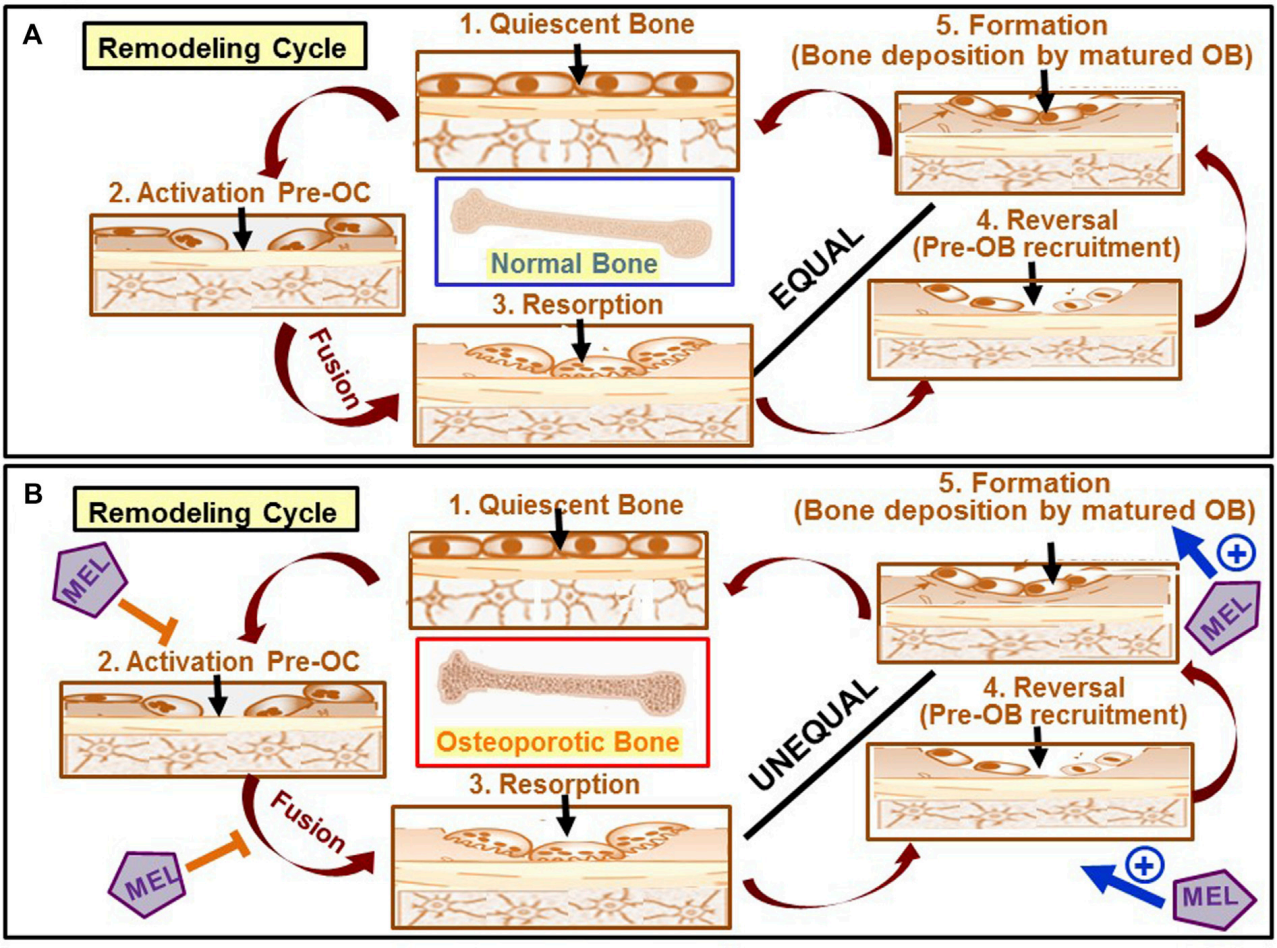
Schematic diagram showing the pathogenesis of osteoporosis and the sites of action of Mel. **(A)** Optimum biomechanical function of bone is achieved by removing old bone and subsequent replacement by new bone through a bone remodeling cycle. In healthy adults, the remodeling cycle begins first by removing old/damaged bones by multinucleated osteoclasts (OC) (stage 3) by the fusion of mononuclear osteoclast precursors such as monocytes and macrophages in the activation phase (stage 2). Pre-osteoblasts formed from MSCs are then recruited to the resorption sites (stage 4), an event known as the reversal stage, followed by their differentiation to osteoblast (OB) that then form new bones (stage 5) to fill the resorption pits. The amount of bone formed in healthy adults is nearly equal to the amount of bone resorbed. **(B)** In women with postmenopausal osteoporosis, while bone resorption becomes exaggerated (indicated as an unequal relationship between stage 3 and stage 4) due to the activation of osteoclasts as a result of a fall in estrogen level, bone formation is concurrently diminished due to a fall in osteoblast differentiation and survival. Mel favorably acts at four stages of the remodeling, inhibits resorption (stages 2 and 3), and promotes bone formation (stages 4 and 5).

As discussed earlier, Mel has diverse actions on bone cells (for summary, see Table 1). The pathogenesis of osteoporosis and the cellular targets of Mel’s action in the context of the bone remodeling cycle are schematically shown in Figure 3.

In MSCs, Mel promotes osteogenic differentiation by multiple mechanisms that include receptor-mediated signaling to stimulate osteogenic genes (Runx2 and osterix), support BMP signaling, upregulate OPG, and downregulate PPARγ expression (Maria et al., 2018; Zhou Y. et al., 2020; Zhang et al., 2021). These effects are achieved by Mel receptor-dependent signaling that involves not only the classical signaling molecules and antioxidant effects of the hormone but also RNAs (miRNAs and lncRNAs) (Li Y. et al., 2019; Wang et al., 2021). As a result, Mel stimulates osteoblast formation and downregulates osteoclast and adipocyte formation. Mel also protects osteoblasts from inflammation and glucose-induced toxicities. In osteoclasts, Mel inhibits differentiation and function by suppressing RANKL-induced ROS production by BMM through the inhibition of NF-κB activation (Maria et al., 2018).

Bone marrow cells produce Mel that appears to protect cells from endogenous and exogenous oxidative stress (Tan et al., 1999). Indeed, in rats, Mel provides a myeloprotective action to bone marrow cells exposed to a cytotoxic drug, aracytin (Anwar et al., 1998). However, the cellular source of Mel in bone marrow is unknown. Future research should identify the Mel positive cells in the bone marrow and explore the paracrine bone-specific effect of this hormone by taking a mouse genetic approach. Moreover, mitochondrial dysfunction inhibits osteogenesis and favors osteoclastic function, together contributing to bone loss during aging. Mitochondria is the major organelle for the generation of free radicals and oxidative stress contributing to aging-related diseases, including osteoporosis (Dobson et al., 2020). Conventional antioxidants such as α-tocopherol, ascorbate, and flavonoids have limited efficacy in mitigating the severity of ROS-related events due to their inability to concentrate in mitochondria and achieve sufficient levels. Mel could serve as an endogenous mitochondria-targeted antioxidant to diminish oxidative stress in bone cells more efficiently and thereby prevent bone loss. Future studies should address the link between the roles of Mel and mitochondrial function in bone cells in the context of aging.

Mammalian aging is associated with the weakening of rhythmic activities, such as circadian sleep/wake rhythms (Kondratova and Kondratov, 2012). Several genes that have diurnal patterns, including Clock, *Bmal1*, Per1, Per2, Cry1, and Rev-erb-α, are expressed in calvaria and long bones of mice. *In vitro*, the expression of these genes has also been reported in osteoblasts and osteoclasts [for a recent comprehensive review, refer to Winter et al. (2021)]. Genetic disruption of clock genes affects bone metabolism. For example, Per2Brdm1 mice (carrying a single mutant Per2) display increased bone formation, and Cry2^−/−^ mice decreased bone resorption (Maronde et al., 2010). In a study on 600 geriatric individuals living in China, 14 tag single nucleotide polymorphisms (SNPs) in seven circadian clock-associated genes, including *clock*, *Per1*, *Per 2*, *Per 3*, *Cry 1*, *Cry 2*, and *MTNR1B* (melatonin receptor 1B) were analyzed. The findings of the study suggest that the Cry 2 rs2292910 and MTNR1B rs3781638 SNPs are predictors of osteoporosis risk in the Chinese population residing in a certain locality (Li et al., 2016). More studies, such as the one on the Chinese geriatric population, are required to better understand the association of circadian genes on osteoporosis risk and the connection of Mel in the process.

As a consequence of the direct effects of Mel in bone cells, it has bone anabolic and anti-resorptive effects in preclinical models of bone loss, including E2-deficient conditions, diabetic animals, and aged animals (for summary, see Table 2). Mel downregulates senescence proteins in bone cells that contribute to its anti-aging effect and hold potential in treating age-related diseases besides osteoporosis. Mel also synergizes the effects of rapamycin on bone, resulting in stimulating bone mass in OVX and aging conditions (Tao et al., 2020). An increase in bone mass by Mel in osteopenic animals in many reports has been shown to accompany an increase in bone strength, which suggests that Mel has a positive effect on bone quality (Taylor et al., 2013; Cakir et al., 2016; Igarashi-Migitaka et al., 2020). Mel also has potential for biomaterial application as when combined with Gel-MA-DOPA to fabricate a composite implantation material, it stimulates osseointegration in the osteoporotic condition through the sustained release of Mel (Xiao et al., 2020).

One of the attractive features of Mel action is its ability to reduce the RANKL/OPG ratio, which has a direct therapeutic implication (Renn et al., 2018). A higher RANKL/OPG ratio over the normal controls promotes bone resorption and is elevated in osteoporosis. A human neutralizing antibody against RANKL (denosumab) is clinically used for the treatment of postmenopausal osteoporosis (Cummings et al., 2009). Because RANK is indispensable for osteoclast formation and function, denosumab completely renders bone resorption inactive. As bone resorption triggers the process of the bone remodeling cycle, which is required for maintaining bone quality, denosumab’s action affects bone quality in the long run. Mel would allow the restoration of the bone remodeling cycle to normal by suppressing RANKL instead of completely inactivating it, thereby preventing overactive bone resorption observed in osteoporosis patients. Another limitation of anti-osteoporosis therapy is the limited window of stimulated bone formation by the osteoanabolic therapies (teriparatide and abaloparatide) (Bhattacharyya et al., 2019). This limitation stems from the stimulatory effect of these drugs on osteoblastic RANKL production that is concurrent with the stimulation of osteoblast survival and differentiation. The stimulatory effect on RANKL production limits the osteoanabolic “window” of these drugs. Mel could be used as a therapy adjunct to teriparatide/abaloparatide to widen the anabolic window of these drugs. Both osteoanabolic therapies, although very effective in increasing vertebral BMD, are not as effective in increasing hip BMD (Bhattacharyya et al., 2019). Therefore, combining Mel with any of these anabolic therapies could provide an additive effect by increasing BMD at both vertebra and hip. Moreover, given the osteogenic effect and anti-resorptive effects of Mel in several preclinical studies, this hormone also holds the potential for standalone anti-osteoporosis therapy. Future human clinical trials could establish Mel as a new class of anti-osteoporosis therapy.

Preclinical studies have shown that the bone anabolic effect of Mel is mediated by MT2R (Sharan et al., 2017). Although the clinically used Mel receptor agonists, agomelatine, and tasimelteon are non-selective between MT1R and MT2R, both have relatively higher affinity to MT2R (Zlotos et al., 2014). Therefore, the likely bone improving effect of these two drugs could be assessed in osteoporosis patients. Moreover, as agomelatine and tasimelteon are used to treat sleep and circadian disturbances, patients using these drugs could be retrospectively assessed for their BMD levels, which would serve as a pointer to their bone mass-promoting effect.

## Glossary

Mel: Melatonin
MT1R: Melatonin receptor 1
MT2R: Melatonin receptor 2
PTH1R: Type 1 receptor for parathyroid hormone
PTHrP: PTH-related protein
MSCs: Mesenchymal stem cells
RANKL: Nuclear factor kappa B ligand
OPG: Osteoprotegerin
GPR 50: G protein-coupled receptor 50
4P-PDOT: 4-Phenyl-2-propionamidotetralin
EGFR: Epidermal growth factor receptor
MnSOD: Manganese superoxide dismutase
SOD: Superoxide dismutase
PPARγ: Peroxisome proliferator-activated receptor gamma
TG: Triglycerides
SMURF1: SMAD-specific E3 ubiquitin protein ligase 1
EGR1: Early growth response 1
BMP: Bone morphogenetic protein
ICAM-1: Intracellular adhesion molecule-1
GC: Glucocorticoid
ER: Endoplasmic reticulum
elF: Translation initiation factors
PERK: Protein kinase RNA-like endoplasmic reticulum kinase
ATF4: Activating transcription factor 4
CHOP: C/EBP homologous protein
SIRT-1: Silent information regulator-1
PBMCs: Peripheral blood mononuclear cells
OVX: Ovariectomy
PNX: Pinealectomy
AN: Anorexia nervosa
GLUT4: Glucose transporter 4
Tph1: Tryptophan hydroxylase
HGF: Hepatocyte growth factor
PTEN: Phosphatase and tensin homolog
P1NP: Procollagen type 1 N-terminal propeptide
CTX-1: Collagen cross-linked C-telopeptide
NLRP3: Nucleotide-binding domain and the leucine-rich repeat pyrin 3 domain
IL: Interleukin
γH2AX: Gamma H2A histone family member X
HG: High glucose
GPx: Glutathione peroxidase
ROS: Reactive oxygen species
Nrf2: Nuclear factor-erythroid factor 2-related factor 2
ZIP-1: Zn^2+^-transporter-1
GelMA-DOPA: Gelatinmethacryloyl-dopamine
HD: Hemodialysis
DXA: Dual-energy x-ray absorptiometry
HR-pQCT: High-resolution peripheral quantitative computed tomography
Per1 and Per2: Period
Cry1 and Cry2: Cryptochrome
SNPs: Single nucleotide polymorphisms
MTNR1B
Melatonin receptor 1B.
